# Training-Dependent Associative Learning Induced Neocortical Structural Plasticity: A Trace Eyeblink Conditioning Analysis

**DOI:** 10.1371/journal.pone.0095317

**Published:** 2014-04-23

**Authors:** Lily S. Chau, Alesia V. Prakapenka, Liridon Zendeli, Ashley S. Davis, Roberto Galvez

**Affiliations:** 1 Psychology Department, University of Illinois at Urbana-Champaign, Champaign, Illinois, United States of America; 2 Beckman Institute for Advanced Science and Technology, University of Illinois at Urbana-Champaign, Champaign, Illinois, United States of America; 3 Neuroscience Program, University of Illinois at Urbana-Champaign, Champaign, Illinois, United States of America; Bilkent University, Turkey

## Abstract

Studies utilizing general learning and memory tasks have suggested the importance of neocortical structural plasticity for memory consolidation. However, these learning tasks typically result in learning of multiple different tasks over several days of training, making it difficult to determine the synaptic time course mediating each learning event. The current study used trace-eyeblink conditioning to determine the time course for neocortical spine modification during learning. With eyeblink conditioning, subjects are presented with a neutral, conditioned stimulus (CS) paired with a salient, unconditioned stimulus (US) to elicit an unconditioned response (UR). With multiple CS-US pairings, subjects learn to associate the CS with the US and exhibit a conditioned response (CR) when presented with the CS. Trace conditioning is when there is a stimulus free interval between the CS and the US. Utilizing trace-eyeblink conditioning with whisker stimulation as the CS (whisker-trace-eyeblink: WTEB), previous findings have shown that primary somatosensory (barrel) cortex is required for both acquisition and retention of the trace-association. Additionally, prior findings demonstrated that WTEB acquisition results in an expansion of the cytochrome oxidase whisker representation and synaptic modification in layer IV of barrel cortex. To further explore these findings and determine the time course for neocortical learning-induced spine modification, the present study utilized WTEB conditioning to examine Golgi-Cox stained neurons in layer IV of barrel cortex. Findings from this study demonstrated a training-dependent spine proliferation in layer IV of barrel cortex during trace associative learning. Furthermore, findings from this study showing that filopodia-like spines exhibited a similar pattern to the overall spine density further suggests that reorganization of synaptic contacts set the foundation for learning-induced neocortical modifications through the different neocortical layers.

## Introduction

It is widely accepted that memory consolidation involves structural plasticity (for review, see [1]). More specifically, dendritic spine modifications have been suggested to play a critical role in learning and memory consolidation. For example, classic studies utilizing general learning and memory tasks, such as environmental enrichment paradigms, have demonstrated robust increased dendritic spine density in the visual [2]–[7], temporal [8] and somatosensory cortex [9] following extended periods of sensory learning. Additionally, other general learning tasks, such as acrobatic training paradigms, have shown increased synaptic density in the motor cortex following various types of motor learning [10]. Together, findings from these general learning studies suggest that structural neuronal plasticity underlies memory consolidation.

Findings from these general learning and memory studies have been pivotal for establishing a now prominent theory that task acquisition and memory consolidation are mediated by the formation of new synaptic connections. Furthermore, based upon these and other learning analyses [11], [12], most agree that synaptic modification in the neocortex underlies memory consolidation. However, this assertion is based upon general learning paradigms, where a subject undergoes multiple different learning events over several days of training, making it difficult to determine the synaptic time course mediating each learning event. Although it is generally accepted that neocortical synaptic modification mediates learning, the time course for neocortical learning induced synaptic changes in response to a single learning event has never been closely examined.

To examine neocortical structural plasticity at different time points during learning, the present study utilized the trace-eyeblink conditioning paradigm. During eyeblink conditioning, subjects are presented with a neutral, conditioned stimulus (CS) (i.e., tone, light, or whisker deflection) paired with a salient, unconditioned stimulus (US) (i.e., air-puff to the eye or a mild periorbital eyeshock) that elicits an unconditioned response (UR) (i.e., eyeblink). With multiple CS-US pairings, subjects learn the CS-US association and exhibit a conditioned response (CR) (i.e., eyeblink) when presented with the CS. In trace conditioning paradigms, there is a stimulus free interval between the CS and the US. Acquisition for this form of conditioning is forebrain-dependent because it requires an intact hippocampus [13]–[17], medial prefrontal cortex [18], [19] and neocortex [20], [21].

To investigate learning-induced neocortical plasticity, the present study took advantage of the whisker barrel system and utilized the trace-eyeblink conditioning paradigm with whisker stimulation as the CS (whisker-trace-eyeblink (WTEB) conditioning). In the rodent whisker system, sensory information from individual whiskers are sent contralaterally to a specific region in layer IV of primary somatosensory cortex (barrel cortex) in a 1∶1 configuration [22]. Prior findings have reported that pre- and post-training lesions of the primary somatosensory cortex impairs WTEB acquisition and retention [20], demonstrating that primary somatosensory cortex is required for both learning and expression of the learned CS-US association. Additionally, previous studies utilizing the WTEB conditioning paradigm have demonstrated that conditioning increases the size of the cytochrome oxidase stained whisker representation for the conditioned whisker barrels in layer IV of primary somatosensory cortex [21], [23], [24]. Furthermore, recent findings from our laboratory demonstrated that WTEB conditioning increases synapsin I expression in conditioned barrels compared to control whisker barrels [23], suggesting that WTEB conditioning induces neocortical synaptic modification. Synapsin I is a phosphoprotein involved with regulating the release of neurotransmitters at the synapse [25], and has been reported to be correlated with synapse number [26]–[29]. Collectively, these studies demonstrate that WTEB conditioning is a neocortical-dependent task that also induces neocortical synaptic modifications making it a suitable paradigm for investigating the timing of learning-induced structural plasticity. The present study used Golgi-Cox staining to examine neocortical spine modifications during and following acquisition for WTEB conditioning.

## Materials and Methods

### Ethics Statement

This study was carried out in strict accordance with the recommendations in the Guide for the Care and Use of Laboratory Animals of the National Institutes of Health. All procedures were performed in accordance with guidelines approved by the University of Illinois at Urbana-Champaign's Institutional Animal Care and Use Committee (Protocol #12153). All surgery was performed under anesthesia, and all efforts were made to minimize suffering.

### Subjects

Thirty-five three-month old male C57BL/6J mice were individually housed under a 12 h light/dark cycle with lights on at 7:00AM, and had access to food and water *ad libitum*.

### Surgery

Mice were surgically implanted with a headpiece necessary for WTEB conditioning, as previously described [30]. Briefly, mice were anesthetized with a ketamine (1 mg/kg, i.p.) and xylazine (6 mg/kg, i.p.) cocktail. Once anesthetized, a plastic strip connector containing two Teflon-coated stainless steel wires and one ground wire was fitted to the head. The Teflon-coated wires were surgically implanted underneath the skin and emerged around the right periorbital region. The headpiece was then secured to the skull with dental acrylic. Mice had at least five days to recover before behavioral training.

### Behavioral Task

**Figure 1:**
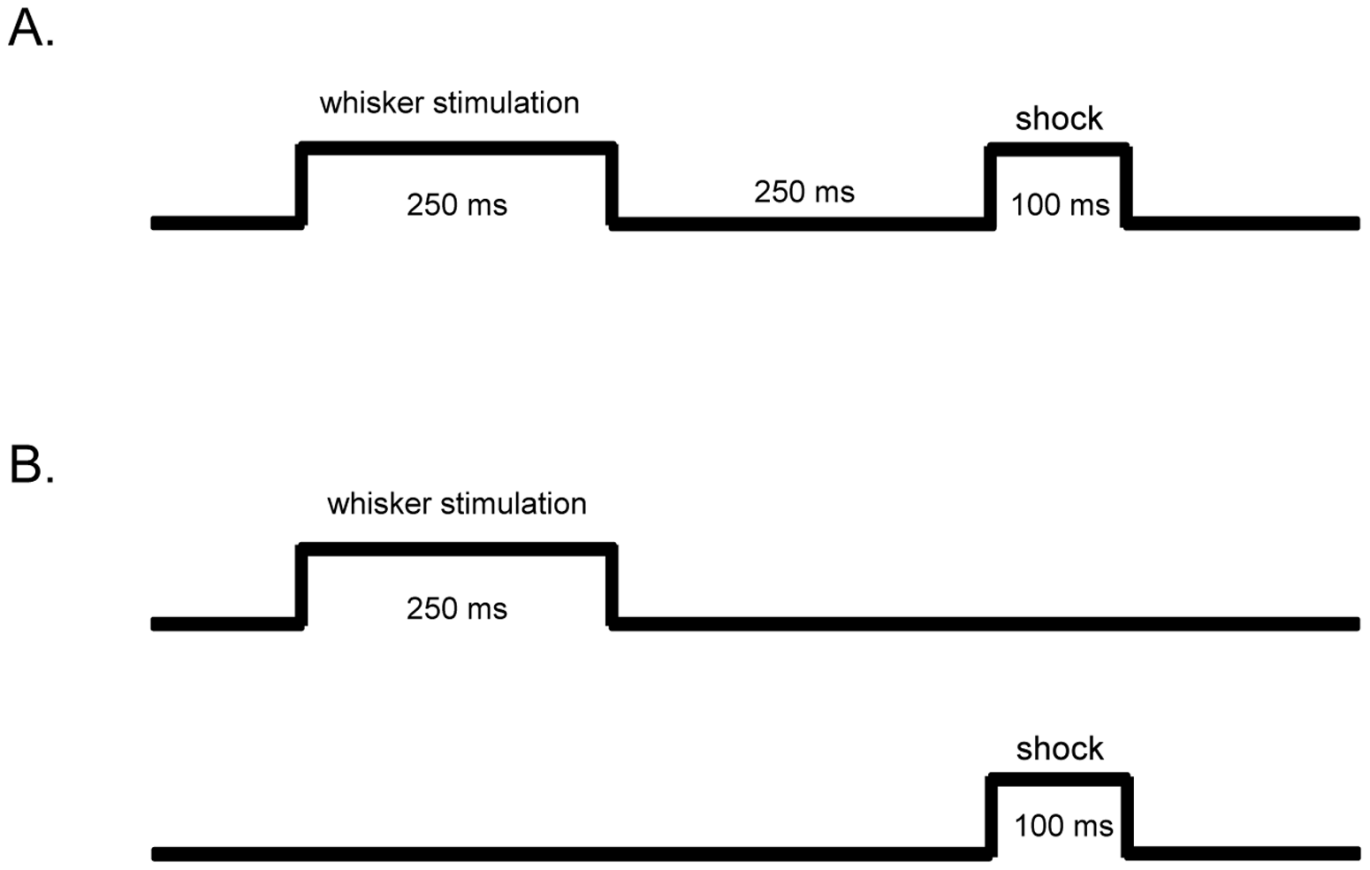
Schematic of conditioning paradigms. Conditioned mice were trained with either a trace-paired conditioning or unpaired conditioning paradigm. (A) Trace-paired-conditioned mice received 250 ms of whisker stimulation (CS), followed by 250 ms of stimulus-free (trace) interval and 100 ms of periorbital shock (US) every trial. (B) Unpaired-conditioned mice randomly received either 250 ms of whisker stimulation or 100 ms of a mild periorbital shock each trial.

Training chambers were standard laboratory cages placed inside a sound-attenuated chamber. Mice were connected to a tether via their headpiece and allowed to move freely in the training chamber for 20 min during habituation. Following habituation, mice were randomly assigned to either trace-paired conditioning (n = 15) or unpaired conditioning (n = 15). A computer running routines written on LabView software delivered all stimuli (whisker stimulation and mild periorbital eyeshock) and collected all behavioral data (eyeblinks). Trace-paired conditioned mice received 250 ms of whisker stimulation delivered via a custom-made whisker stimulator [30], 250 ms of stimulus-free (trace) interval followed by 100 ms of periorbital shock (0.1 to 1 mA periorbital square wave shock, 60 Hz, 0.5 ms pulses) (Figure 1A). Trace-paired conditioned mice were given 30 trials per session with a 45 s mean intertrial interval (ITI) ranging from 30 to 60 s. An optic sensor placed in front of the right eye was used to monitor eyelid closure. Using information from the optic sensor, a CR was defined as a 4 standard deviation change in voltage from baseline occurring within 35 ms of CS onset [14], [31], [32]. Unpaired-conditioned mice randomly received either a whisker stimulation or periorbital shock each session with a 22 s mean ITI (varied randomly between 15 to 30 s) (Figure 1B). Note that unpaired-conditioned mice (stimulation-controls) are termed pseudo-conditioned mice in some studies. All trace-paired conditioned and unpaired-conditioned mice received one conditioning session consisting of 30 trials per day. Mice in the trace-paired conditioning group were further randomly assigned to either the acquisition (ACQ), learned (LRD) or over-trained (OT) group. ACQ mice were trained until three-CRs were exhibited out of five consecutive trials, LRD mice were trained until four-CRs were exhibited out of five consecutive trials and OT mice were trained until two sessions of four-CRs exhibited out of five consecutive trials (note that these are referred to as ‘days to criterion’). Unpaired-conditioned (unpaired) mice were randomly yoked to trace-paired conditioned mice (unpaired-ACQ; unpaired-LRD; unpaired-OT), and collected at the same time. Naïve mice (n = 5) did not undergo any surgery or eyeblink conditioning, and their brains were collected at the same time as all of the other mice.

### Golgi Processing

Subjects were given an overdose of sodium pentobarbital 1 h following the last conditioning session and transcardially perfused with 0.1 M phosphate buffered saline (PBS) before their brains were processed for Golgi-Cox staining [33], [34]. Briefly, the neocortex was dissected, flattened and placed into a standard Golgi-Cox solution for 55 days. Once impregnated, the flattened neocortices were embedded in 10% celloidin and sectioned at 80 µm. The flattened neocortices were then stained with methylene blue for neocortical barrel visualization, mounted onto slides and coverslipped.

### Data Analysis

**Figure 2:**
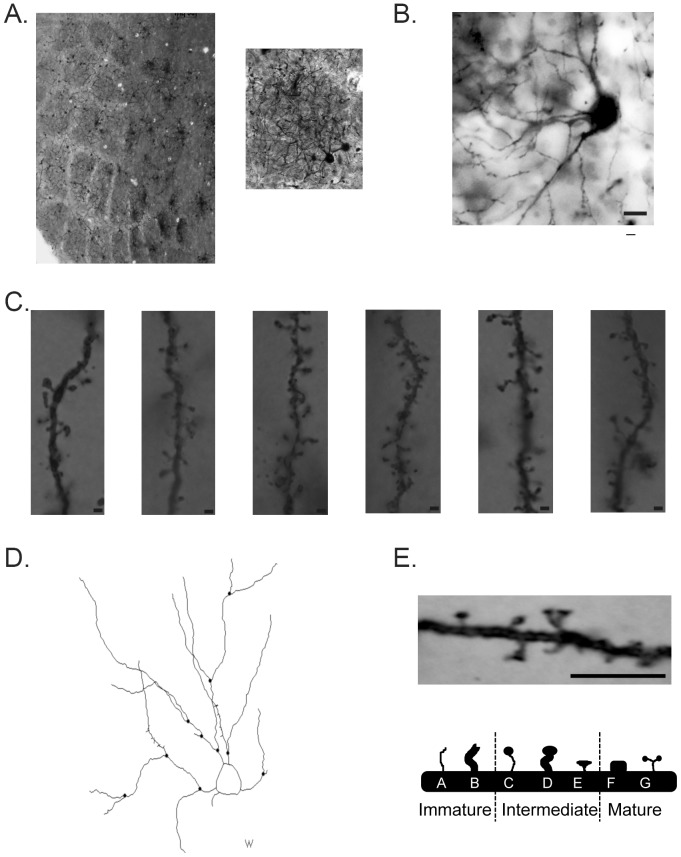
Representative photomicrographs of Golgi-Cox stained stellate neurons and Spine Morphology Types. (A) Representative photomicrographs depicting Golgi-Cox and methylene blue co-staining at 2.5× magnification (left) and 20× magnification (right). Note that only spiny stellate neurons in the inner one-third of the barrel wall were examined. (B) Photomicrograph depicting Golgi-Cox staining at 60× magnification. Scale bar  = 10 µm. (C) Photomicrographs depicting Golgi-Cox staining at 100× magnification for representative dendritic segments. Scale bar  = 1 µm. (D) Neurolucida tracing of a spiny stellate neuron. (E) Representative photomicrographs of dendritic spine morphologies (top). Scale bar  = 5 µm. Depiction of immature, intermediate and mature spine types (bottom).

Neocortical barrels were localized using the methylene blue staining at 2.5× magnification with a Zeiss AxioImager A1 light microscope (Figure 2A). Once a neocortical barrel was localized, spiny stellate neurons located in the inner one-third of the neocortical barrel wall were digitally traced at 100× magnification using Neurolucida Software (MicroBrightField, Williston, VT, USA; Figure 2D). Note, only sections with visible neocortical barrels were analyzed. Scholl sphere analysis was also conducted using the same software, with each ring 10 microns apart. For the neuronal bifurcation ratio (NR), the total number of bifurcating branches (all branch orders) was divided by the sum of all bifurcating and normal ending branches (all branch orders). Overall spine density and spine densities for the different spine morphologies were examined on secondary and tertiary branches exhibiting more than ten spines. Only secondary and tertiary branches were examined due to the limited observations of dendritic spines on primary dendrites. Spines were characterized into four types of morphologies: filopodia-like, thin with bulbous head (bulbous), stubby and branched (similar to previously described neocortical dendritic spine morphologies [35]; see [36] for review). More specifically, filopodia-like spines were thin spines that had a neck and head that were the same size, bulbous spines had a head that was wider than the neck, stubby spines resembled a box and branched spines had more than one head. These spine types were further categorized into immature (filopodia-like), intermediate (bulbous) and mature (stubby and branched) (Figure 2E).

### Statistics

Behavioral analysis was conducted with a two-way ANOVA. Analyses of overall spine density and spine densities of the different spine morphologies (i.e., immature, intermediate and mature) were conducted with a one-way ANOVA. When appropriate, follow-up post hoc analyses used Fisher's LSD criterion for significance and all comparisons were considered statistically significant if p<0.05 as calculated by SigmaPlot (Version 11.0, Systat Software, Chicago, IL, USA) and SPSS (Version 14.0, IBM Software, Armonk, NY, USA).

## Results

### Behavioral Analysis

**Figure 3:**
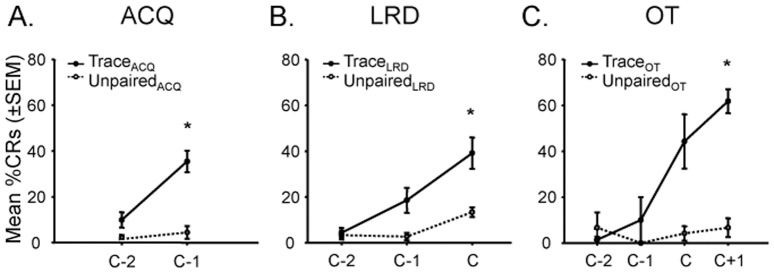
All trace-paired conditioned mice learned the WTEB conditioning task, in contrast to their respectively yoked unpaired-conditioned mice. (A) Mean percent conditioned response (CR) (±SEM) for ACQ mice each session until ACQ criterion (C-1  =  day of ACQ criterion; C-2  =  day before ACQ criterion. (B) Mean percent conditioned response (CR) (±SEM) for LRD mice each session until LRD criterion (C =  day of LRD criterion; C-1  =  day before LRD criterion; C-2  =  two-days before LRD criterion). (C) Mean percent conditioned response (CR) (±SEM) for OT mice each session until OT criterion (C+1  =  day of OT criterion; C =  day before OT criterion; C-1  =  two-days before OT criterion; C-2  =  three-days before OT criterion). All trace-paired conditioned mice (ACQ, LRD and OT) exhibited a significant increase in WTEB conditioning performance compared to unpaired-conditioned mice, and in comparison to their baseline performance. *p<0.05.

A two-way within subjects ANOVA demonstrated a significant difference between groups (F_(5,52)_ = 13.26, p<0.05), days to criterion (F_(2,52)_ = 14.89, p<0.05) and interaction between groups and days to criterion (F_(10,52)_ = 2.17, p<0.05; Figure 3). Post hoc analyses using Fisher's LSD criterion for significance indicated that OT mice (M = 61.83; SD = 11.70) performed significantly better than OT-yoked-unpaired mice (M = 6.67; SD = 8.17), LRD mice (M = 39.12; SD = 18.13) and ACQ mice (M = 35.45; SD = 8.15). Furthermore, LRD mice (M = 39.12; SD = 18.13) performed significantly better than LRD-yoked-unpaired mice (M = 13.33; SD = 4.71), and ACQ mice (M = 35.45; SD = 8.15) performed significantly better than ACQ-yoked mice (M = 6.67; SD = 0.00). On average, it took ACQ mice 2.25 days to reach the ACQ requirement, LRD mice 2.67 days to reach the LRD requirement and OT mice 3.80 days to reach the OT requirement (Figure S1). Together, these results demonstrate that ACQ, LRD and OT mice, unlike their respectively yoked unpaired-conditioned mice, learned the WTEB conditioning task.

### Golgi Analyses

There were no significant differences detected between unpaired-ACQ, unpaired-LRD and unpaired-OT mice for any of the subsequent golgi analyses, so the data was combined into a single respective group (unpaired). Additionally, there were no significant differences in overall spine densities or spine densities of the different spine morphologies examined between secondary and tertiary branches within each treatment group, so these were collapsed into their respective groups as well. Also, note that spines were further classified into immature (filopodia-like), intermediate (bulbous) and mature (stubby and branched) spines.

### Overall Spine Density

**Figure 4:**
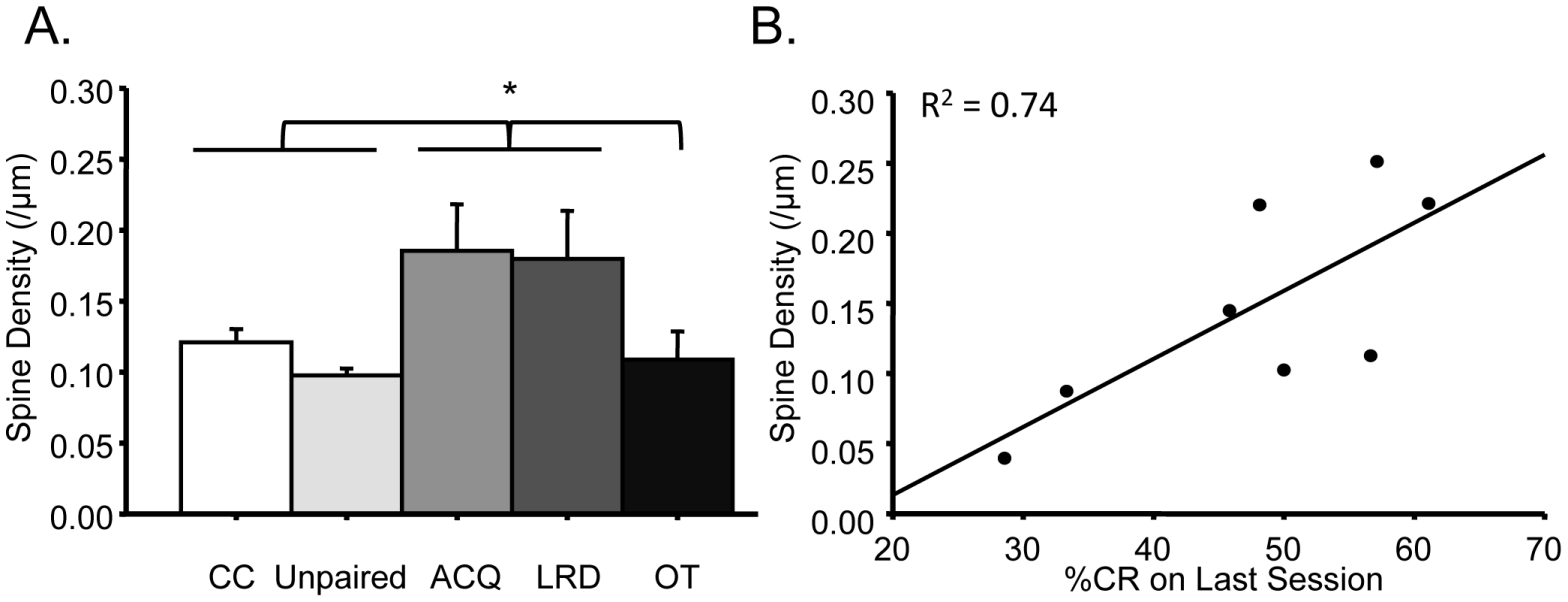
Increased spine proliferation during memory formation for WTEB conditioning. (A) ACQ and LRD mice exhibited greater overall spine density compared to cage-control, unpaired and OT mice. (B) Overall spine density of ACQ and LRD mice are positively correlated to WTEB conditioning performance on last session. *p<0.05.

A one-way ANOVA demonstrated a significant difference between groups (F_(4,45)_ = 4.89, p<0.05; Figure 4A). Post hoc analyses using Fisher's LSD criterion for significance indicated that ACQ mice exhibited significantly greater spine density (M = 0.18; SD = 0.08) compared to cage-control mice (M = 0.12; SD = 0.03), unpaired mice (M = 0.10; SD = 0.02) and OT mice (M = 0.11; SD = 0.05). Additionally, LRD mice (M = 0.18; SD = 0.10) exhibited significantly greater spine density compared to cage-control mice (M = 0.12; SD = 0.03), unpaired mice (M = 0.10; SD = 0.02) and OT mice (M = 0.11; SD = 0.05). There were no significant differences in overall spine density between ACQ and LRD mice. Overall spine density of OT mice (M = 0.11; SD = 0.05) were significantly different from ACQ mice (M = 0.18; SD = 0.08) and LRD mice (M = 0.18; SD = 0.10), but were not significantly different from unpaired mice (M = 0.10; SD = 0.02) or cage-control mice (M = 0.12; SD = 0.03), suggesting that over-training returns overall spine density to control levels. Further analyses demonstrated that performance on the last WTEB conditioning session for ACQ and LRD mice were significantly correlated to their overall spine density, R^2^ = 0.74, p<0.05; Figure 4B).

### Immature, Intermediate and Mature Spine Densities

#### Immature Spines (Filopodia-Like)

**Figure 5:**
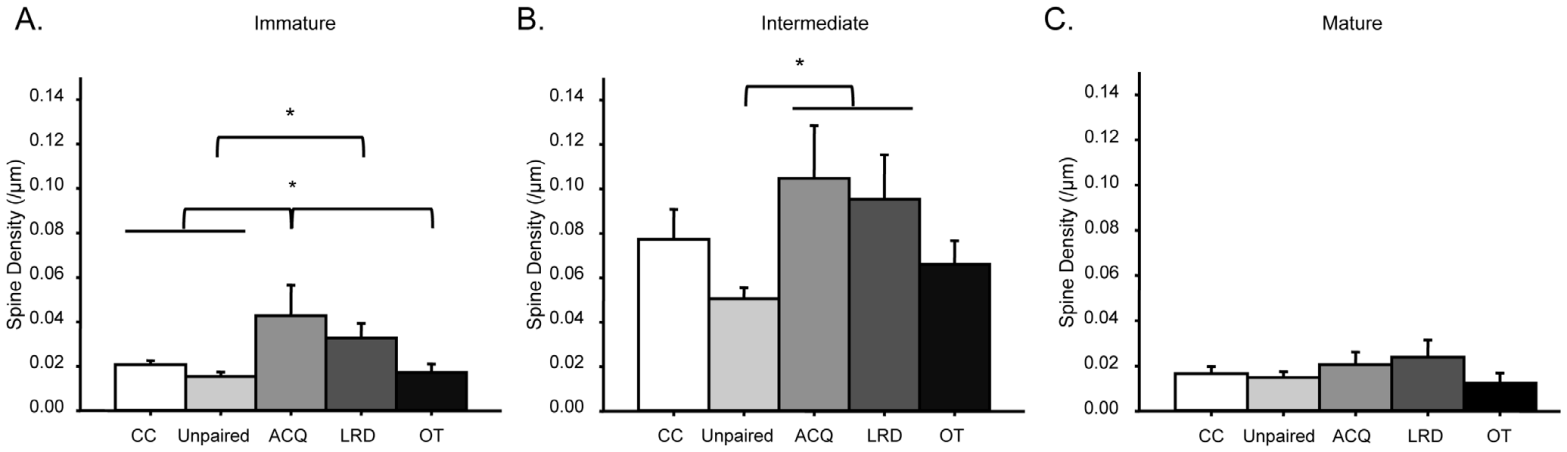
Spine density of immature, intermediate and mature spines at different time points of WTEB conditioning. (A) ACQ mice exhibited significantly more filopodia-like spines than cage-control, unpaired and OT mice. LRD mice exhibited significantly more filopodia-like spines than unpaired mice. (B) ACQ and LRD mice exhibited significantly more bulbous spines than unpaired mice. (C) LRD mice exhibited a trend for greater spine density of mature spines compared to OT mice. *p<0.05.

A one-way ANOVA demonstrated a significant difference between groups (F_(4,45)_ = 4.65, p<0.05; Figure 5A). Post hoc analyses using Fisher's LSD criterion for significance indicated that ACQ mice exhibited greater density of filopodia-like spines (M = 0.04; SD = 0.03) compared to cage-control (M = 0.02; SD = 0.006), unpaired (M = 0.02; SD = 0.009) and OT mice (M = 0.017; SD = 0.01). Additionally, LRD mice exhibited greater density of filopodia-like spines (M = 0.03; SD = 0.02) compared to unpaired mice (M = 0.02; SD = 0.009). There were no differences detected between ACQ and LRD mice.

#### Intermediate Spines (Bulbous)

A one-way ANOVA demonstrated a significant difference between groups (F_(4,43)_ = 3.00, p<0.05; Figure 5B). Post hoc analyses using Fisher's LSD criterion for significance indicated that both ACQ (M = 0.11; SD = 0.06) and LRD mice (M = 0.10; SD = 0.06) exhibited significantly greater density of bulbous spines than unpaired mice (M = 0.05; SD = 0.02).

#### Mature Spines (Combination of Stubby and Branched Spines).

A one-way ANOVA did not detect any significant differences in spine density of mature spines between any of the groups (Figure 5C). However, a pre-planned comparison indicated a trend (p = 0.056) for LRD mice (M = 0.024; SD = 0.03) to exhibit greater spine density of mature spines than OT mice (M = .012; SD = 0.02).

### Dendritic Branching

No significant differences in dendritic material or dendritic branching were detected between any of the groups from the scholl sphere and bifurcation ratio analyses, respectively (see Figure S2).

## Discussion

Classic findings utilizing general learning and memory paradigms demonstrating increased dendritic material and dendritic spine density in the neocortex have strongly suggested that memory consolidation involves neocortical structural plasticity [2]–[7]. However, the time course for these neocortical anatomical modifications during a specific learning task has not been closely examined. The present study utilized the forebrain-dependent trace associative learning paradigm, WTEB conditioning, to examine neocortical structural plasticity at different time points during learning.

Analyses from the current study demonstrated that layer IV spiny stellate neurons in ACQ and LRD mice exhibited a greater spine density compared to control, unpaired and OT mice (Figure 4A). These findings suggest that neocortical spine proliferation facilitates acquisition of associative learning tasks, consistent with previous reports from general learning and memory paradigms [4], [5], [8]. Additional analyses demonstrated a significant correlation between overall spine density and WTEB conditioning performance of ACQ and LRD mice (Figure 4B), offering further support that spine proliferation plays a role during task acquisition and memory formation. These findings are also consistent with previously proposed mechanisms of learning and memory [36] and more recent reports of increased dendritic spine formation in the motor cortex during early training of various motor-learning tasks [37]. Together, these findings paralleling previous analyses demonstrate that neocortical spine proliferation occurs during acquisition and further suggest that remodeling of neocortical networks play an essential role during memory formation.

Further analyses found that with over-training, the overall spine density in layer IV of primary somatosensory cortex returned to control levels (Figure 4A), suggesting a transient increase in overall neocortical spine density during learning. Though these findings appear inconsistent with the previously discussed analyses from general learning and memory tasks [2]–[7], the time course of structural plasticity during learning in many of these general learning and memory paradigms precludes analyses of underlying time-specific mechanisms. Furthermore, this transient increase in spine density is consistent with hippocampal analyses of spine density following spatial learning tasks. For example, studies have found that hippocampal spine density increases and returns to baseline levels after learning of hippocampal-dependent tasks such as the morris water maze [38], [39] and avoidance learning [40]. Similarly, other findings have reported a hippocampal time-dependent increase in spine density following long-term potentiation [41], one of the most common molecular models of learning and memory. However, to our knowledge, the current study is the first to demonstrate a training-dependent transient increase in spine density during associative learning in the neocortex. Together, these findings suggest that learning, at least in some brain regions, results in a transient increase in spine density facilitating synaptic reorganization.

This training-dependent transient increase in neocortical spine density could have resulted from a number of different anatomical mechanisms. Experience-dependent plasticity studies have reported pruning of newly formed spines in the neocortex following sensory learning [42]. In contrast, motor learning tasks, such as forelimb reaching tasks, have demonstrated pruning of more mature spines following learning [37]. The drop in overall spine density in layer IV spiny stellate neurons in primary somatosensory cortex following WTEB conditioning could be due to either mechanism. However, irrespective of which spine population is being selectively removed, these findings suggest that learning in layer IV results in reorganization of primary thalamic synaptic input.

Findings from this study also demonstrated significantly increased spine density of filopodia-like spines in ACQ mice compared to controls, unpaired and OT mice (Figure 5A), paralleling the overall spine density analysis previously discussed. These findings suggest that filopodia-like spines are contributing to the increase in overall spine density and that proliferation of these immature spines facilitates associative learning task acquisition. These analyses are consistent with previous reports proposing that thin, filopodia-like spines play a critical role in learning (for review, see [36]). More specifically, previous studies have demonstrated increased density of thin spines in the cerebellum following complex motor learning [43]. Additionally, studies have reported that the density of thin spines in the prefrontal cortex correlates with learning performance in aging subjects [44], further suggesting that thin spines are fundamental for learning. These analyses, in conjunction with our findings, suggest that the proliferation of filopodia-like spines is important for memory formation and plays a key role in initial neocortical rewiring during learning.

Further spine morphology analyses demonstrated that bulbous spines were significantly increased in ACQ and LRD mice compared to unpaired mice (Figure 5B). Previous studies have demonstrated that immature spines (i.e., filopodia-like spines) transition into intermediate spines (i.e., bulbous spines) following general sensory learning. In particular, experience-dependent plasticity studies utilizing whisker deprivation have reported the maturation of newly formed thin spines to bulbous spines following general sensory learning [45]. Furthermore, previous studies have reported synapse formation of newly formed spines four days after proliferation [42], similar to the time frame of when LRD mice were collected (see Figure S1), suggesting that these intermediate spines are able to communicate with other neurons and thus become further integrated into the neocortical network. Collectively, findings from these studies suggest that acquisition-induced filopodia-like spines are transitioning into bulbous spines during memory formation, and that these bulbous spines are important for rewiring of the neocortical network during learning.

There were no significant differences detected in mature spine densities between any of the groups, but there was a trend for increased spine density of mature spines in LRD mice compared to OT mice (p = 0.056) (Figure 5C) suggesting that the intermediate spines are transitioning into mature spines, as previously reported following sensory learning [45]. This trend is consistent with previous studies reporting increased branched spines in the hippocampus [46], [47] and striatum [48] following associative learning and environmental enrichment, respectively. However, the lack of a significant overall effect in mature spine morphologies in our analyses could be due to the amount of training the animals underwent. In the current study, animals were only trained one day beyond reaching learning criterion. In many prior analyses, animals were trained for several days beyond criterion [49]. Although this could account for the observed differences, more recent findings in the prefrontal cortex have also found no significant correlation between the number of mature spines and learning ability [44], suggesting that the number of mature spines in the neocortex, unlike the hippocampus and striatum, do not correlate with learning.

To our knowledge, there have been few examinations of neocortical plasticity at different time points during learning for a more specific learning paradigm, such as trace associative learning. Classic learning and memory studies have suggested the importance of structural plasticity, especially dendritic spine proliferation, for memory consolidation, but few have closely examined neocortical dendritic plasticity at different time points during that learning process. Findings from this study demonstrate that forebrain-dependent trace associative learning induces training-dependent neocortical spine proliferation. Furthermore, our analyses of the different spine morphologies suggest that in the neocortex, filopodia-like spines proliferate during memory formation. Based upon previously discussed findings, these immature spines then transition into intermediate and mature spines, resulting in rewired neocortical input. Together with previous findings, these analyses suggest that the neuronal mechanisms underlying learning are a training-dependent process resulting in the reorganization of synaptic contacts beginning at the site of primary thalamic input to the neocortex, layer IV. Furthermore, these findings suggest that this reorganization of synaptic contacts would set the foundation for learning-induced neocortical modifications through the different neocortical layers. Subsequent analyses are needed to determine the implications of these synaptic reorganizations on neuronal connections throughout all six neocortical layers.

## Supporting Information

Figure S1 **Mean training sessions for trace-paired conditioned mice to reach ACQ, LRD or OT.** (A) Mean percent conditioned response (CR) (±SEM) for ACQ mice each session. The arrow indicates the mean training session it took ACQ mice to exhibit three-CRs out of five consecutive trials. (B) Mean percent conditioned response (CR) (±SEM) for LRD mice each session. The arrow indicates the mean training session it took LRD mice to exhibit four-CRs out of five consecutive trials. (C) Mean percent conditioned response (CR) (±SEM) for OT mice each session. The arrow indicates the mean training session it took OT mice to exhibit four-CRs out of five consecutive trials for two sessions.
(TIF)

Figure S2 **No significant difference in dendritic material or dendritic branching between groups.** (A) Scholl sphere analysis did not detect any differences between groups. (B) Bifurcation ratio analysis did not detect any differences between groups.
(TIF)
