# Supplementary figures and images for: Training-Dependent Associative Learning Induced Neocortical Structural Plasticity: A Trace Eyeblink Conditioning Analysis
Source: PLoS One. 2014 Apr 23;9(4):e95317. doi: 10.1371/journal.pone.0095317 (PMC3997347; doi:10.1371/journal.pone.0095317)

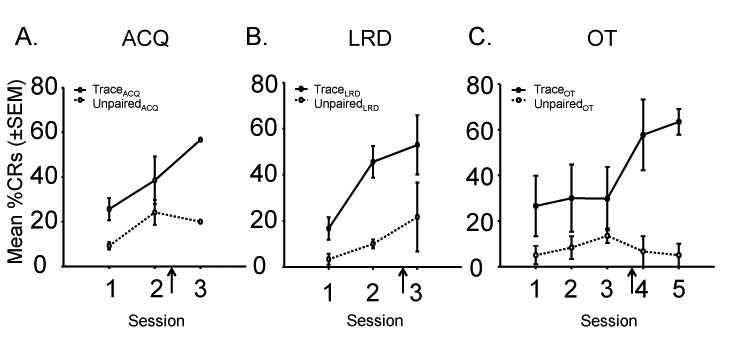

Supplement: Figure S1 — Mean training sessions for trace-paired conditioned mice to reach ACQ, LRD or OT. (A) Mean percent conditioned response (CR) (±SEM) for ACQ mice each session. The arrow indicates the mean training session it took ACQ mice to exhibit three-CRs out of five consecutive trials. (B) Mean percent conditioned response (CR) (±SEM) for LRD mice each session. The arrow indicates the mean training session it took LRD mice to exhibit four-CRs out of five consecutive trials. (C) Mean percent conditioned response (CR) (±SEM) for OT mice each session. The arrow indicates the mean training session it took OT mice to exhibit four-CRs out of five consecutive trials for two sessions. (TIF) [file pone.0095317.s001.tif]

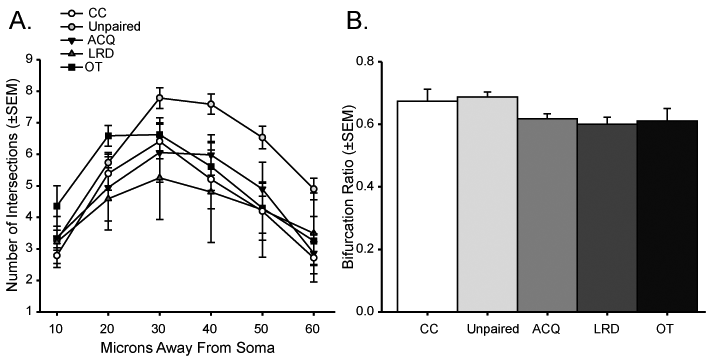

Supplement: Figure S2 — No significant difference in dendritic material or dendritic branching between groups. (A) Scholl sphere analysis did not detect any differences between groups. (B) Bifurcation ratio analysis did not detect any differences between groups. (TIF) [file pone.0095317.s002.tif]
